# Deciphering Repertoire of B16 Melanoma Reactive TCRs by Immunization, In Vitro Restimulation and Sequencing of IFNγ-Secreting T Cells

**DOI:** 10.3390/ijms22189859

**Published:** 2021-09-12

**Authors:** Anna V. Izosimova, Diana V. Yuzhakova, Valeria D. Skatova, Lilia N. Volchkova, Elena V. Zagainova, Dmitry M. Chudakov, George V. Sharonov

**Affiliations:** 1 Institute of Experimental Oncology and Biomedicine, Privolzhsky Research Medical University, 10/1 Minin and Pozharsky Sq., 603950 Nizhny Novgorod, Russia; annizosimova@mail.ru (A.V.I.); yuzhakova-diana@mail.ru (D.V.Y.); liliya.barbashova@gmail.com (L.N.V.); ezagaynova@gmail.com (E.V.Z.); chudakovdm@mail.ru (D.M.C.); 2 Lobachevsky State University of Nizhny Novgorod, 23 Gagarin Ave., 603950 Nizhny Novgorod, Russia; 3 Shemyakin-Ovchinnikov Institute of Bioorganic Chemistry RAS, 16/10 Miklukho-Maklaya, 117997 Moscow, Russia; backward607@gmail.com; 4 Department of Molecular Technologies, Institute of Translational Medicine, Pirogov Russian National Research Medical University, 1 Ostrovityanova, 117997 Moscow, Russia

**Keywords:** immunotherapy, tumor-specific T lymphocytes, T cell receptor (TCR), B16F0 melanoma, antigen-specific T cells

## Abstract

Recent advances in cancer immunotherapy have great promise for the treatment of solid tumors. One of the key limiting factors that hamper the decoding of physiological responses to these therapies is the inability to distinguish between specific and nonspecific responses. The identification of tumor-specific lymphocytes is also the most challenging step in cancer cell therapies such as adoptive cell transfer and T cell receptor (TCR) cloning. Here, we have elaborated a protocol for the identification of tumor-specific T lymphocytes and the deciphering of their repertoires. B16 melanoma engraftment following anti-PD1 checkpoint therapy provides better antitumor immunity compared to repetitive immunization with heat-shocked tumor cells. We have also revealed that the most error-prone part of dendritic cell (DC) generation, i.e., their maturation step, can be omitted if DCs are cultured at a sufficiently high density. Using this optimized protocol, we have achieved a robust IFNγ response to B16F0 antigens, but only within CD4+ T helper cells. A comparison of the repertoires of IFNγ-positive and -negative cells shows a prominent enrichment of certain clones with putative tumor specificity among the IFNγ+ fraction. In summary, our optimized protocol and the data provided here will aid in the acquisition of broad statistical data and the creation of a meaningful database of B16-specific TCRs.

## 1. Introduction

Immunotherapy has great promise in cancer treatment. One of the most successful clinical approaches is the release of antitumor immunity by checkpoint therapy. It uses monoclonal antibodies to block inhibitory checkpoints, which are normally dedicated to the prevention of exaggerated autoimmunity. Cytotoxic T lymphocyte-associated protein 4 (CTLA-4) and programmed cell death protein 1 (PD-1) are two checkpoints that have been approved by the FDA and successfully used in clinics [1,2,3]. Despite the success in cancer treatment and the obvious potential of checkpoint inhibitor immunotherapy, its effectiveness does not exceed 10–30% for most common types of cancer [4].

It is known that checkpoint therapy not only activates a tumor-specific response, but also stimulates a response to autoantigens and persistent infections [5]. Previously, we obtained data suggesting that the effectiveness of immunotherapy is determined by the stability of anti-PD1-elicited clonal T cell expansions [6], which could reflect the ratio of these types of response. However, it is almost impossible to understand the proportion of tumor-specific responses against the background of the systemic activation of immunity caused by checkpoint therapy. Therefore, the crucially important goal is to identify tumor-specific clones on nonspecific background activation. This will provide distinctive information on the magnitude and mechanism of specific antitumor responses.

In addition, the identification of tumor-specific cells is a key step in another promising approach to immunotherapy: adoptive cell therapy [7,8]. Obtaining tumor-specific cells for in vitro expansion is the most challenging step in this therapeutic approach. Alternatively, tumor-specific T cells can be generated by gene cloning of the TCR, specifically recognizing tumor antigens if the latter have been identified [9].

An extensive search for antigen-specific cells in various pathologies began in the 1980s after the nature of their specificity and the antigen-driven clonal expansion had been discovered [10,11]. Tumor-specific cells for mice and humans were successfully generated in several studies [12,13,14,15]. However, actual protocols of tumor-specific lymphocyte isolation for adoptive cell therapy [8,15,16,17] are much more sophisticated than they were in those pioneer studies, indicating limited applicability of the latter.

In the majority of the studies, especially in earlier ones, the antigen-specific responses were evaluated by proliferation according to [^3^H]-thymidine incorporation that provides no data on quantities and TCRs of specific cells. Moreover, the data on isolation and/or identification of tumor-specific lymphocytes for wild-type B16 melanoma are also extremely limited in spite of the high value of this cancer model [18,19]. In Shitaoka et al., B16-specific CD8+ T cells were isolated by the sorting of CD137+PD1+CD8+ cells from tumor-infiltrating lymphocytes (TILs) and selecting enriched clones [20]. This demonstrates the power of repertoire analysis for deciphering tumor-reactive TCR. Yet, there is an obvious lack of actual data on B16-specific TCRs, especially for CD4+ cells.

In this work, we have optimized a protocol for deciphering the TCR repertoire of B16F0 melanoma-reactive cells. The first important step was the generation of antitumor immunity in mice by the repetitive inoculation of tumor cells and selection of tumor-free mice. Most efficiently, antitumor immunity was established when mice were treated with anti-PD-1 antibodies. Specific cells were enriched from splenocytes by their restimulation with tumor antigens in vitro and the selection of activated cells by IFNγ capture assay. Several parameters of the in vitro stimulation protocol were optimized, namely the generation of antigen presenting cells, antigen preparation and loading, T cell isolation, in vitro stimulation conditions and the sorting of live activated cells for TCR repertoire analysis.

## 2. Results

### 2.1. Generation of Antitumor Immunity in Mice

In our experience with more than 200 mice, inoculation of 10^5^–10^6^ B16F0 tumor cells (day 0) reproducibly gave rise to palpable tumors on days 5–20 in 90% of mice (data not shown). The second inoculation of tumor cells in the resting 10% of mice gave nearly the same tumor engraftment efficiency. This indicates that it is unlikely that failure of engraftment is due to the formation of antitumor immunity in these settings.

**Figure 1:**
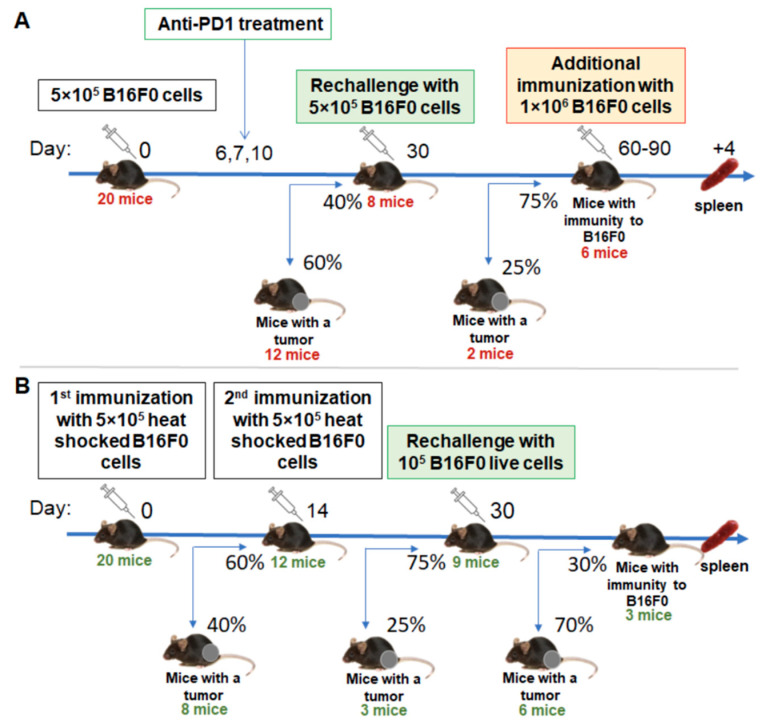
Scheme for generation of mice with immunity to melanoma B16F0. Induction of antitumor immunity by anti-PD1 therapy (**A**) and by two immunizations with heat-shocked tumor cells (**B**). Tumor-free mice are presented on the main axis, while mice that fail to reject tumors are presented on the bottom and were excluded from the experiment. Mice count and percentages after each tumor inoculation are presented.

In order to boost antitumor immunity, we have used two approaches: anti-PD1 checkpoint blockade and immunization with tumor cells compromised by heat shock. A triple injection of therapeutic antibodies starting on day 6 gave rise to a significantly increased percentage of tumor-free mice to 40% (Figure 1A). Rechallenging these mice with a second injection of tumor cells confirmed the formation of antitumor immunity, since only 25% of the tumors were engrafted. 

With the second approach, we used a suspension of tumor cells that were subjected to heat shock for 1 h at 43 °C. We stained these cells with propidium iodide and AnnexinV and analyzed them with flow cytometry 2h after heat shock. We found that 12–15% of them were viable (*n* = 2), 8–10% were apoptotic and the resting cells were dead (data not shown). Vaccination with this tumor cell vaccine gave 40% and 25% tumor engraftment after the first and second inoculations, respectively (Figure 1B). Further rechallenge with live tumor cells indicated that the antitumor immunity in these mice has been established but with much less inefficiency compared to the anti-PD1 group.

### 2.2. T Cell Isolation

**Figure 2:**
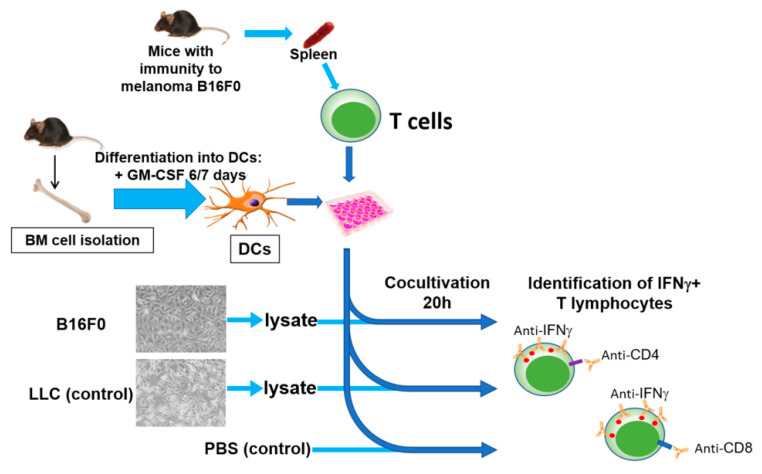
General scheme of in vitro restimulation of T lymphocytes with tumor antigens, including isolation of T lymphocytes from the spleens of mice with antitumor immunity, isolation of bone marrow (BM) cells with subsequent differentiation into DCs, obtaining a lysate/microparticles of tumor cells, in vitro stimulation of T cells with DCs loaded with tumor lysate, and identification and selection of tumor-specific T lymphocytes.

In order to identify tumor-specific clones, we isolated and reactivated, in vitro with tumor antigens, T cells from spleens of tumor-free mice. The overall scheme of the in vitro restimulation is shown in Figure 2.

For the isolation of T lymphocytes from spleens, we tested negative and positive magnetic separation. Both methods have advantages and disadvantages. Positive selection allows for a higher percentage of enrichment of the target population to be obtained, while negative selection does not cap the surface of the target cells with antibodies, and thus better preserves their physiological functions.

**Figure 3:**
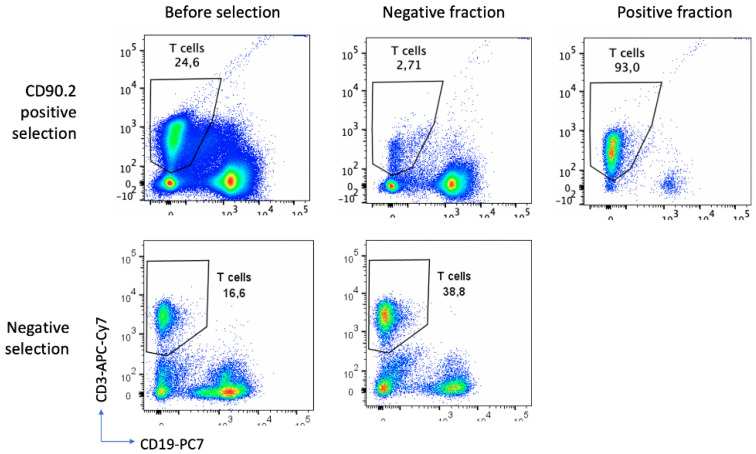
Comparison of the purity of lymphocyte isolation by positive selection with anti-CD90.2 MicroBeads (upper row) and negative selection (lower row). The first column shows the original cells without selection, the second, the negative fraction after selection, and the third, the positive fraction after selection. The histograms of the distribution of cells in accordance with the expression of the marker of T cells (CD3) and B cells (CD19) are presented. The area and percentage of T cells are marked on the histograms.

Analysis of the resulting fractions showed that negative selection allows for only a moderate enrichment of the T cells (from 17% to 39%), while positive selection with anti-CD90.2 MicroBeads provides a purity of more than 90% (Figure 3). Therefore, in further experiments, T cells were isolated using positive selection. From a single spleen, we usually obtained 2.5–4.5 × 10^6^ T lymphocytes.

### 2.3. Generation of Antigen Presenting Cells

To select the optimal conditions for the generation of DCs, we investigated the effect of various cultivation conditions, such as the concentration of cells in the well of the plate, the composition of the nutrient medium, and the duration of cell cultivation.

We tested two cell densities per well: 1 × 10^6^ cells/mL (1 or 0.5 × 10^6^ per well of a 24– or 48–well plate, respectively) and 2 × 10^6^ cells/mL (2 or 1 × 10^6^ per well of a 24– or 48–well plate, respectively).

**Figure 4:**
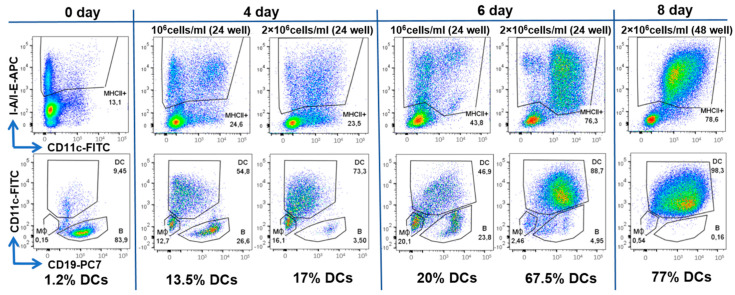
Analysis of the cultured BM cells. Flow cytometry histograms of cells according to the expression level of MHCII (I-A/I-E), CD11c and CD19 are shown. Data are presented for cells before cultivation (day 0) and after 4, 6 and 8 days of cultivation, as well as for two cell densities: 1 × 10^6^ cells/mL and 1 × 10^6^ cells/mL. For each timepoint and concentration, two histograms are shown: on the upper ones, MHCII+ cells are gated, which are displayed on the lower histograms showing expression of CD19 and CD11c expression by MHCII+ cells. On the latter, the areas of DCs, B cells and macrophages (MΦ) are marked, as well as their percentages.

It was found that, at a lower cell density, the enrichment of DCs is weaker. The fraction of MHCII+ cells does not exceed 44%, and the percentage of CD11+ (DCs) from MHCII+ cells does not exceed 55%. The possible explanation for this is deficiency in cell–cell contacts and paracrine autostimulation. At a higher cell density, we observed a significantly greater enrichment of MHCII+ and DCs, with the percentage of the target populations increasing over time. The best results were observed on days 7–8 of cultivation: the percentage of MHCII + was about 78%, and most of them (98%) were CD11c+. Thus, the DCs fraction was 77% of the total cell number (Figure 4).

**Figure 5:**
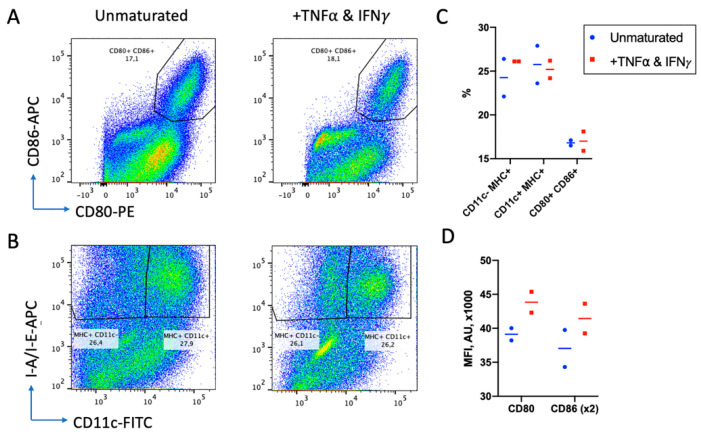
Effect of TNF-α and IFNγ on DC maturation. After 6 days of differentiation, DC cells were either untreated or stimulated overnight with TNFα and IFNγ. (**A**) Histograms representing the amount of CD80+/CD86+ double positive cells. (**B**) Histograms representing percentage of MHC+CD11c- macrophages and MHC+CD11c+ DCs. (**C**) Percentages of cells of different phenotypes in two replicas. (**D**) Expression level of CD80 and CD86 measured as MFI (in arbitrary units, AU) of staining with corresponding antibodies. Two replicas are presented.

Cell survival and morphology were better when 50 mM β-mercaptoethanol was added to the culture medium. Differentiation into DCs also depended on granulocyte macrophage colony stimulating factor GM-CSF and IL-4, favoring the generation of MHCII+ CD11c+ CD19- DC cells over MHCII+ CD11c- CD19- macrophages. The addition of a maturation cocktail of TNFα and IFNγ on day 6 resulted in the slight increase in CD80+ and CD86+ expression level (mean fluorescence intensity, MFI, Figure 5D), but not the DC fraction, and compromised DC survival (Figure 5C).

Thus, for further experiments, BM cells were cultured for 7 days on 24-well plates at a density of 2 × 10^6^ cells/mL with the GM-CSF, IL-4 and without a maturation stage.

### 2.4. Antigen Loading and In Vitro Stimulation of T Cells

**Figure 6:**
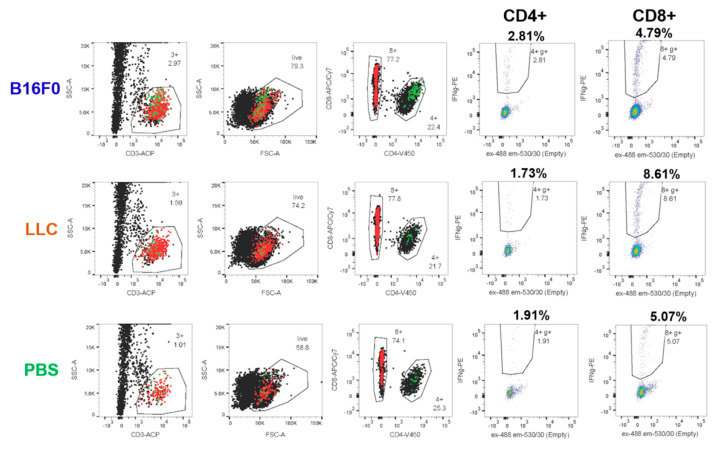
Detection of tumor-specific cells after 44h stimulation with either DCs loaded with tumor cell lysate (B16F0 or LLC) or unloaded DCs (PBS). Gating scheme is shown from left to right: CD3+ T cells (3+), live CD3+ cells (live), subdivided into CD4+ (4+) and CD8+ (8+) cells. The fourth and fifth columns show gates for activated IFNγ+ cells among CD4+ (4+ g+) and CD8 + (8+ g+). The percentages of cells in each gate are shown above the graphs. Activated CD4+ and CD8+ cells are overlaid on the histograms in the first three columns in green and red, respectively.

Tumor-specific T cells were detected according to their reactivation in response to tumor antigens in vitro. Lymphocyte activation was measured on the basis of IFNγ+ production (Figure 6) that was found to give a better signal to the background ratio compared to cell surface activation markers CD25, CD134 and CD137 (data not shown). Here, we used intracellular cell staining (ICS) for the detection of IFNγ+ cells.

**Figure 7:**
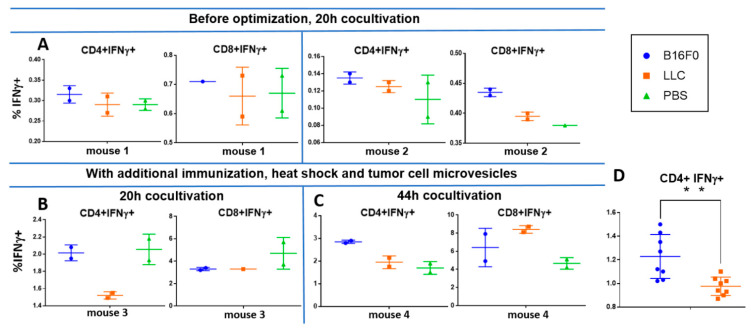
Percentage of IFNγ+ cells among CD4+ and CD8+ cells under different conditions of stimulation by DCs and tumor antigens. Cells cocultured in the presence of B16F0 lysate, LLC lysate and without lysate (PBS) were compared. Each condition was analyzed in duplicate for each mouse. (**A**) Spleen T lymphocytes of mice, which were finally inoculated with the tumor more than 1 month before the experiment. To prepare tumor antigens, tumor cells were not subjected to heat shock and microvesicles were not used. (**B**,**C**) T lymphocytes from spleens of mice, which were restimulated with B16F0 cells 4 days before the experiment. Tumor lysates were prepared after heat shock and combined with microparticles from the conditioned medium of these. Cells were cocultured either for 20 h (**A**,**B**) or for 44 h (**C**). (**D**) Normalized IFNγ+ cell percentages among CD4+ lymphocytes for all settings (4 mice, 8 samples). ** Student’s *t*-test *p* < 0.005.

According to the initial protocol, T cells isolated by magnetic activated cell sorting (MACS) were cocultured with DCs at a 1:1 ratio for 20 h in the presence of a B16F0 tumor cell lysate. The wells with the addition of irrelevant Lewis lung carcinoma (LLC) tumor lysate or the wells without the lysate with the addition of phosphate-buffered saline (PBS) were used as controls. Without lysate, we detected a background of 0.1–0.3% and 0.4–0.7% activated IFNγ+ cells among CD4+ and CD8+ T cells, respectively. Stimulation with a nonrelevant LLC lysate did not increase the percentage of activated IFNγ+ cells. Cultivation in the presence of B16F0 lysate led to an increase in the activation of CD4 + and CD8 + cells by ~ 0.02 and ~0.05 percentage points, respectively (Figure 7A). This indicates the ability to detect tumor-specific cells, albeit with quite a low frequency.

To increase the percentage of tumor-specific cells, we used several approaches. First, we additionally immunized mice by subcutaneous injection of B16F0 cells 4 days before T-cell isolation from the spleens. In addition, we enhanced the immunogenicity of the lysate by heat shock [21]. For this, the cells were heat-shocked by 1h incubation at 42 °C. Microvesicles from a conditioned medium of stressed cells were concentrated with 150 kDa cutoff membrane (see Section 4.6), mixed with freeze–thawed lysate of the same cells and used to load DCs. Due to the limited number of mice with anti-tumor immunity, we applied several modifications at the same time, so one cannot discern their individual effects.

With these optimizations, we registered an increase in the background percentage of activated IFNγ+ cells up to 2% and 4–5% among CD4+ and CD8+ cells, respectively, after 20 h coculture. However, cocultivation with tumor lysate and microvesicles gave a lower percentage of activation in the case of both LLC and B16F0 (Figure 7B). This can be explained by the presence of certain immunosuppressive factors in the lysate and/or microparticles. However, the B16F0 lysate already gave a 0.5 percentage points increase in the activation of CD4+ cells compared to the LLC lysate (Figure 7B).

Increasing the co-cultivation time to 44 h resulted in a decrease in background activation in samples without lysate to 1.7% IFNγ+ cells among CD4+ cells and remained the same among CD8+ cells (Figure 7C). At the same time, the percentage of IFNγ+ among CD4+ cells co-cultured with B16F0 lysate was significantly higher than the rest of the samples and amounted to approximately 2.8%. This effect can be explained by the rapid depletion of immunosuppressive factors and diminishing of its effect over longer coculture periods. At the same time, it became possible to reliably register 1% of B16F0-specific CD4+ T lymphocytes from the spleen against the background of nonspecific activation (Figure 7C).

The limited number of mice and the large number of variable parameters in the experiments did not allow for us to obtain statistical estimates for any particular experiment. In order to evaluate the statistical significance of the differences between B16F0 and LLC lysates, we normalized and combined all experiments. Normalization was applied on a per-mouse basis. The average percentage of IFNγ+ cells in LLC probes was taken for a unit. With this normalization, B16F0 lysate gave a significantly higher (p < 0.005) percentage of IFNγ+ cells for eight samples (four mice) compared to the LLC lysate, but only for CD4+ cells (Figure 7D).

### 2.5. Repertoire Analysis of IFNγ+ Cells

**Figure 8:**
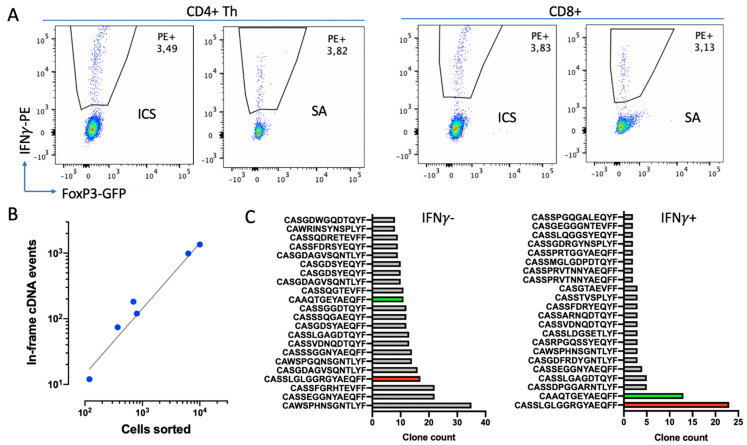
Isolation and analysis of repertoire of live IFNγ+ cells. (**A**) Comparison of IFNγ+ cells detection by ICS and SA techniques. Histograms represent cells according to IFNγ+ staining and FoxP3-GFP expression (FoxP3+ Treg cells are gated out from CD4+ subset) for CD4+ Th cells (left panel) and CD8+ cells (right panel). (**B**) Efficiency of repertoire extraction depending on the number of sorted cells. Number of in-frame TCRβ cDNA reads for different numbers of sorted cells are presented. (**C**) Repertoire truncate for 10^4^ IFNγ- (left) and 700 IFNγ+ cells. Clone counts for the top 22 TCR clones are shown. CDR3 regions presented as amino-acid sequences. Two clones enriched in IFNγ+ subset are highlighted in green and red.

Dealing with limited-cell-number unique molecular identifier (UMI)-based repertoire sequencing from the RNA starting material produces more reliable results than DNA-based methods [22]. Therefore, we aimed to isolate live IFNγ+ cells by means of SA. A comparison of the percentages of IFNγ+ cells detected by secretion assay (SA) and ICS from a single mouse showed consistent results (Figure 8A).

Next, we analyzed whether this protocol provides a sufficient material amount and quality of mRNA to extract the TCR repertoire. We extracted and analyzed TCRβ-chain repertoires of 10^2^–10^4^ IFNγ+ or IFNγ− sorted CD4+ Th cells from three samples (Figure 8B,C). Here, we obtained 167 ± 55 (Mean±SD, *n* = 6) TCRβ in-frame cDNA events per 1000 sorted cells within the entire range of sorted cells’ quantities (Figure 8B). This protocol of repertoire extraction appeared to be less efficient compared to freeze-free techniques based on cDNA synthesis directly in lysis solution and RNA purification with TRIzol [23]; however, it provides much higher flexibility and reliability.

A preliminary comparison of repertoires of IFNγ+ and IFNγ− cells revealed their substantial differences. The repertoire of IFNγ+ cells was poorer compared to the repertoire of IFNγ- cells, with a couple of clones being highly enriched (Figure 8C). Although these clones were also found in IFNγ-, they were not so prominent. This indicates that the elaborated technique allows us to enrich certain clones with putative tumor specificity.

## 3. Discussion

Here, we have optimized a protocol for the selective stimulation of T lymphocytes with mouse melanoma B16 antigens. In mice, this task is simpler than in humans because linear tumor and mice models have nearly constant antigens and MHCs. Nonetheless, these results can be reproduced or used in other experiments with the corresponding mouse strain and/or tumor model. This can be useful because the mechanisms of antitumor immunity are far from being resolved, even in mice, in spite of impressive advances in the immunotherapy of human cancers. For example, there are still almost no data on TCR specificity for mouse tumor antigens (https://vdjdb.cdr3.net/, accessed on 20 July 2021). We chose melanoma B16 as one of the most common mouse tumor models [18,19].

In order to enhance antitumor immunity, a strong artificial antigen (particularly ovalbumin or viral proteins) is often expressed in tumor cells [19,24]. This skews immune response to a certain pathway in a predictable manner, but on the other hand makes it less physiological. Here, we used the unmodified B16F0 murine melanoma tumor line that appeared to be almost non-immunogenic, without additional stimuli. We found that checkpoint therapy with anti-PD1 antibodies produces a stable antitumor immunity in 30% of mice. Of note, the response to anti-PD1 therapy in human melanoma patients is also around 30% [4], indicating the relevance of the chosen tumor model for the study of anti-PD1 therapy.

During the development of immunotherapy approaches, researchers avoid dealing with bulk tumor mass as a source of antigens because of its complexity, low efficiency and low reproducibility. Instead, individual peptide neoantigens are preferable [18,25,26]. In this work, we used a tumor lysate as a source of antigens. Compared to synthetic peptides or RNAs bearing single mutation (neoantigens), tumor lysate may contain other possible antigens such as tumor-associated antigens, antigens formed as a result of indels, reading frame shifts, alternated slicing and translation [27]. Neoantigens are thought to be the primary immune target [28]; however, other types of antigens can also significantly contribute to the cancer immunogenicity and have been efficiently targeted in several clinical trials [27,29].

Here, we used tumor lysate as a source of antigens to broaden the immune response and make it more physiological but, on the other hand, it complicates the control and reproduction of experimental conditions and requires optimization. In particular, we found that, after a heat shock and in the presence of microparticles, the tumor lysate has immunosuppressive activity that declines within 2 days. This is in agreement with other studies that demonstrated the immunosuppressive activity of tumor-derived exosomes [30,31,32].

We used heat-shocked tumor cells due to the notion that lysate of unstressed cells does not induce noticeable immune response or even can be immunosuppressive [33,34]. Heat shock is known to enhance antigenicity and immunogenicity by a bunch of mechanisms [35,36]. Here, we fractionated lysed cells and conditioned medium (see Section 4.6) in order to exclude crude cell mass and to ensure the absence of viable tumor cells. However, it is hard to achieve highly reproducible substances with fractionation. Combining heat shock with irradiation and lysis prevents the appearance of live tumor cells in the preparation and can be used to give a reproducible substance for vaccination and/or DC loading [37,38].

In this work, we have revealed a significant CD4+ Th response to tumor lysate, while the cytotoxic response of CD8+ lymphocytes is not prominent. A possible reason for this is the use of naive DCs that first require licensing from CD4, which delays CTL response [39]. Moreover, Kreiter et al. have also shown that CD4+ response is crucial for effective anti-B16 immunity in mice after vaccination with tumor antigens, while CD8+ cells can be depleted without a noticeable effect on tumor rejection [18].

DCs have a crucial function in antitumor immunity and may guide immune response in various or even opposite directions [40]. Keeping this in mind, we carefully optimized the protocol for DC differentiation. We found that cell density is crucial for effective DC differentiation, the parameter that is usually neglected in the literature. We also found that, after efficient differentiation, the additional maturation step is redundant. If maturation was performed with lipopolysaccharide, we obtained a high background activation of lymphocytes (data not shown), while the use of TNFα and IFNγ did not have a significant effect on the expression of MHCII and key costimulatory molecules CD80 and CD86. The maturation of human monocyte-derived DCs with TNFα, Il1β and prostaglandin E2 also significantly compromises the viability of DCs, but also increases expression of CD80 and CD86 approx. 2.5 fold (data not shown). In our experience with human DCs, the maturation step is also most error-prone among the whole procedure and requires thorough control of all reagents and incubation times.

## 4. Materials and Methods

### 4.1. Tumor Cell Culture

A B16F0 murine melanoma cell line was used in the study. B16F0 cells were cultured in Dulbecco′s Modified Eagle′s Medium (DMEM) with 10% fetal bovine serum (FBS, Gibco, Amarillo, TX, USA), 0.06% L-glutamine, 50 units/mL penicillin and 50 μg/mL streptomycin. The cell culture was maintained in a CO_2_-incubator at 37 °C and 5% CO_2_. To obtain the tumor model, B16F0 cells were cultured on 25 mm^2^ plates and collected for the injection by adding 1 mL of trypsin-EDTA solution (0.25%) per flask for 5 min at 37 °C. In some experiments, we used heat-shocked tumor cells for the immunization and loading of tumor antigens into APCs. For these purposes, cells were detached with trypsin-EDTA, washed and resuspended in PBS at a final concentration of 5 × 10^6^/mL in PBS. This suspension was heated to 43 °C for 1 h and used without wash.

### 4.2. Tumor Model and Immunotherapy

The experiments were carried out on 40 transgenic C57Bl/6-FoxP3-EGFP mice (kindly provided by Alexander Rudensky, Sloan Kettering Institute, New York, NY, USA). Transgenic mice were generated against the C57Bl/6 genetic background, by knocking in the chimeric construct of eGPF subcloned into the first exon of FoxP3 gene [41].

Tumors were generated by subcutaneous (s.c.) injection of 5 × 10^5^ B16F0 melanoma cells suspended in 100 μL PBS into the mouse leg. To obtain mice with prominent clonal T cell antitumor immunity, two approaches were compared: anti-PD-1 immunotherapy and repetitive immunization with suspension of heat-shocked tumor cells. The immunotherapy was started on the 6th day after tumor cell injection when tumor nodules were not yet palpated (a tumor was considered palpable when the tumor volume reached 10 mm^3^). Mice were intraperitoneally injected by 250 μg of anti-PD-1 (Clone RMP1-14, Bio X Cell, Lebanon, NH, USA) on the 6th, 7th and 10th days after tumor injection (Figure 1A).

In another setting, mice were immunized twice with 5 × 10^5^ heat-shocked B16F0 cells with a 14-day interval (Figure 1B). 

Tumor-free mice were re-challenged with 5 × 10^5^ B16F0 melanoma cells into the opposite limb after they had been tumor-free for 30 days to confirm the formation of immunological memory. If mice were tumor-free for 30–60 days after tumor rechallenge, they were considered to have stable antitumor immunity. Indicated mice were additionally re-stimulated with 5 × 10^5^ B16F0 melanoma cells 4 days before T-cell isolation in order to increase the percentage of activated tumor-specific cells. Mice were euthanized and splenic T cells were isolated.

All animal experiments were carried out in accordance with the National Institutes of Health Guide for the Care and Use of Laboratory Animals (NIH Publications No. 8023, revised 1978). The experimental protocol was approved by the Ethical Committee of the Privolzhsky Research Medical University Academy, Russia (EC #6, granted 17 April 2019)

### 4.3. T-cell Isolation by Magnetic Separation

Mouse spleens were collected and homogenized using a gentleMACS automatic tissue homogenizer (Miltenyi Biotec, Bergisch Gladbach, Germany) in buffer (PBS pH 7.2, 0.5% BSA and 2 mM EDTA), with the addition of 10 µg/mL DNase I (Roche Diagnostics, Indianapolis, IN, USA) to avoid cell clumping. The resulting cell suspension was passed through a 70 µm cell strainer and erythrocytes were lysed for 5 min with erythrocyte lysis buffer (ELB) (Buffer EL, Qiagen, Hilden, Germany). All further cell manipulations were performed on ice. Magnetic cell labeling and separation were performed according to manufacturer protocol. We tested two different types of magnetic separation to collect T cells. Positive selection was carried out with anti-CD90.2 MicroBeads (Miltenyi Biotec). Negative selection was carried out with a mixture of biotinylated antibodies (CD11b, CD11c, CD19, CD45R, CD49b, CD105, anti-MHCII, Ter-119) (Miltenyi Biotec) against red blood cells and most leukocyte sets, excluding T cells. In both cases, cells were separated with LS Columns and the QuadroMACS magnet (Miltenyi Biotec), and both negative and positive fractions were collected and analyzed by flow cytometry.

### 4.4. Isolation of Murine Bone Marrow and Generation of DCs

The general scheme for in vitro stimulation and selection is represented in Figure 2. For antigen presentation, we used bone marrow (BM)-derived dendritic cells. BM was processed as described elsewhere [42]. Briefly, femur and tibia bones were isolated, both ends of the bones were cut and bone marrow was flushed with 2 mL of PBS using a 1 mL insulin syringe with a 27G×½ needle (the bones should appear white once all the marrow has been expelled) and collected in the centrifuge tubes. The resulting suspension was centrifuged at 300× *g* 5 min, filtered through a 70 µm cell strainer and erythrocytes were lysed for 5 min with ELB.

To generate DCs, BM cells were cultured in 24- or 48-well plates at various concentrations (1 × 10^6^ cells/mL and 2 × 10^6^) in RPMI-1640 (Gibco) with 10% FBS, 0.06% L-glutamine, 50 units/mL penicillin, 50 μg/mL streptomycin and 20 ng/mL GM-CSF and IL-4. For DC maturation, we used 50 ng/mL TNFα and 50 ng/mL IFNγ (both from Miltenyi Biotec).

Approximately 1/2 of the volume of the cultivation medium was replaced with a fresh one at the time of its acidification to pH < 6.5. The frequency of changing the medium depended on the cell concentration. Thus, at a concentration of 2 × 10^6^ cells/mL, the medium was changed on days 2, 4, and 6, and at a concentration of 1 × 10^6^ cells/ mL only on days 4 and 6.

Bone marrow cells were collected at different timepoints and analyzed by flow cytometry.

### 4.5. Flow Cytometry

Stained cells were analyzed on a BD FACSAria III cell sorter (BD Biosciences, San Jose, CA, USA). Data were analyzed using FlowJo software (BD/Treestar, Ashland, OR, USA). FoxP3+ regulatory T lymphocytes were identified on the basis of EGFP fluorescence. 

### 4.6. Preparation of Tumor Cell Lysate

B16F0 melanoma cells were used as a source of tumor antigens, and LLC cells were used as a control to determine the level of nonspecific stimulation (Figure 2). The cells were collected from the plates, centrifuged at 200× *g* 5 min, resuspended in PBS at a concentration of 2–3 × 10^6^ cells/mL and lysed by 5–6 cycles of rapid freezing in liquid nitrogen (−196 °C) and heating in a water bath (37 °C). Cell debris was removed from the resulting suspension by centrifugation.

In a series of experiments to increase the immunogenicity of lysate tumor, cells were exposed to heat shock before lysis. The plates with cells were placed in a thermostat at 41 °C for 1 h. Then, the culture medium and the tumor cells were collected, cells were lysed, and the culture medium was centrifuged at 1000× *g* 10 min to remove the cell debris. The supernatant was concentrated in 20 mL concentration tubes with a permeable membrane for proteins with a molecular weight of less than 150 kDa (Pierce Concentrators 150K MWCO, Thermo Scientific, Waltham, MA, USA) to harvest heat-shock-induced microvesicles. The protein concentrations in the lysate and in the microvesicle preparation were determined using the colorimetric method based on bicinchoninic acid (Pierce BCA Protein assay kit, Thermo Scientific). 

### 4.7. In Vitro Restimulation of T Cells

T cells and tumor cell lysate or tumor cell lysate with heat-shock-induced microparticles were added to DCs on the 7th day of DCs cultivation at a final concentration of T cells 2.5–3 × 10^5^ cells/mL. Lysate was added at a final concentration of 200 μg/mL for B16F0 cells and 100 μg/mL for LLC cells. The higher protein concentration in the case of the B16F0 cell lysate was used to compensate for the prevalence of melanin in this culture that constitutes up to 40% of the total amount of protein [43]. Microvesicels were added to a final concentration of 50 μg/mL of protein. PBS was added to the cells as a control.

T lymphocytes were co-cultured with DCs in the presence of tumor lysate for 20 or 44 h, while the contents of the wells were mixed by pipetting at intervals of 4 h during the day and 8 h at night.

### 4.8. Intracellular Staining

For the detection of IFNγ+ cells by the ICS method, 5 μg/mL of brefeldin-A (GolgiPlug, BD Biosciences) was added to inhibit the formation of vesicular transport from the Golgi complex. After 4 h, the contents of each well of the culture plate were harvested, washed, fixed, permeabilized and stained with antibodies CD3-APC, CD4-V450, CD8-APC/Cy7 (all from BD Biosciences) and IFNγ-PE (Miltenyi Biotec) according to the recommendations of the manufacturer (Inside Stain Kit, Miltenyi Biotec).

### 4.9. Isolation of Live IFNγ Secreting cells

Since reactive IFNγ+ cells are limited, it is preferable to use RNA as the starting material for TCR sequencing. Furthermore, the DNA material is also damaged in fixed cells, impeding the efficient, multiplex-based TCR library preparation. Thus, independently of the RNA or DNA start, sorting the living T cells is highly preferable for TCR repertoires analysis. To this end, here, we used an IFNγ SA (Miltenyi Biotec) to isolate live IFNγ+ T-cells by FACS [12]. For these experiments, we used spleen T cells from mice immunized twice with heat-shocked B16F0 cells. After coculture with DCs, T cells were covered by an IFNγ catch reagent and stained with an IFNγ detection antibody (PE) following counterstaining with CD3-APC, CD4-V450 and CD8-APC/Cy7 antibodies (all from BD Biosciences), and then sorted by FACS.

### 4.10. TCR Repertoire Analysis

TCR sequencing was performed starting from RNA. Cells were sorted directly in 200 μL RLT lysis buffer (Qiagen). Lysed cells were stored for up to 6 months in the RLT buffer at −20 °C. TCR repertoires were obtained with a UMI-based 5′ RACE kit (MiLaboratory, Moscow, Russia) and sequencing on Illumina MiSeq in 150 + 150 nt paired-end mode, as described previously [23,44]. UMI-based clustering of raw sequencing reads was performed with MiNNN software (https://github.com/milaboratory/minnn, accessed on 20 July 2021), and repertoires were extracted using MiXCR [45,46].

## 5. Conclusions

Protocols for the detection of antigen-specific cells are usually presented as a brief final summary, with some crucial details omitted. Consequently, these protocols cannot always be easily reproduced or modified. Here, we presented a detailed consideration of nearly all steps of the protocol, with an evaluation of their different parameters. This allows one to use these data to modify their own protocols and to adopt them for other immunological tasks. For example, to reveal distinct antigen specificities, we modified it for use with certain antigenic peptides. We believe that the acquisition of specificities to certain B16 antigenic peptides and a comparison with TCR repertoires of cells that respond to tumor lysate and overall tumor-infiltrating lymphocytes will provide a clue to decoding antitumor response in this model.
